# Assessing the role of transmission chains in the spread of HIV-1 among men who have sex with men in Quebec, Canada

**DOI:** 10.1371/journal.pone.0213366

**Published:** 2019-03-06

**Authors:** Luc Villandré, Aurélie Labbe, Bluma Brenner, Ruxandra-Ilinca Ibanescu, Michel Roger, David A. Stephens

**Affiliations:** 1 Department of Epidemiology, Biostatistics, and Occupational Health, McGill University, Montréal, Québec, Canada; 2 Department of Decision Sciences, HEC Montréal, Montreal, Québec, Canada; 3 McGill AIDS Centre, Lady Davis Institute, Jewish General Hospital, Montreal, Québec, Canada; 4 Centre de Recherche du Centre Hospitalier de l’Université de Montréal (CRCHUM), Montreal, Québec, Canada; 5 Département de microbiologie, infectiologie et immunologie, Université de Montréal, Montreal, Québec, Canada; 6 Department of Mathematics and Statistics, McGill University, Montréal, Québec, Canada; University of Cincinnati College of Medicine, UNITED STATES

## Abstract

**Background:**

Phylogenetics has been used to investigate HIV transmission among men who have sex with men. This study compares several methodologies to elucidate the role of transmission chains in the dynamics of HIV spread in Quebec, Canada.

**Methods:**

The Quebec Human Immunodeficiency Virus (HIV) genotyping program database now includes viral sequences from close to 4,000 HIV-positive individuals classified as Men who have Sex with Men (MSMs), collected between 1996 and early 2016. Assessment of chain expansion may depend on the partitioning scheme used, and so, we produce estimates from several methods: the conventional Bayesian and maximum likelihood-bootstrap methods, in combination with a variety of schemes for applying a maximum distance criterion, and two other algorithms, DM-PhyClus, a Bayesian algorithm that produces a measure of uncertainty for proposed partitions, and the Gap Procedure, a fast non-phylogenetic approach. Sequences obtained from individuals in the Primary HIV Infection (PHI) stage serve to identify incident cases. We focus on the period ranging from January 1st 2012 to February 1st 2016.

**Results and conclusion:**

The analyses reveal considerable overlap between chain estimates obtained from conventional methods, thus leading to similar estimates of recent temporal expansion. The Gap Procedure and DM-PhyClus suggest however moderately different chains. Nevertheless, all estimates stress that longer older chains are responsible for a sizeable proportion of the sampled incident cases among MSMs. Curbing the HIV epidemic will require strategies aimed specifically at preventing such growth.

## Introduction

In Canada, Men who have Sex with Men (MSMs) are especially at risk of contracting HIV [1]. In Montreal, Québec, for instance, HIV prevalence in that risk group could be as high as 13% [2]. Analyses of HIV genetic sequences provided by MSMs who participated in the Quebec HIV genotyping program have revealed the existence of many separate transmission chains, and suggested an association between chain length and incidence: 42% of MSMs infected between 2012 and 2015 belonged to a transmission chain comprising 20 or more known cases, compared to 13% [3] between 2004 and 2007.

Highly Active Antiretroviral Therapy (HAART) has been successful in substantially suppressing viremia within the diagnosed population, making late transmission of the virus a lot less common, and consequently, early transmission has been increasingly driving the epidemic [4]. Recently-infected individuals are much more likely to transmit because of high viral load, viral homogeneity, and immature immune response, potentially leading to *transmission cascades*, consecutive transmission events happening in a short time span [5]. Those cascades result in the expansion of existing chains and may point to a higher proportion of early transmissions. However, their contribution to incidence is hard to measure accurately due to their estimation being very sensitive to the sampling time of infected individuals [6].

Montreal has become a UNAIDS Fast Track City in May 2017, and the only way to reach the 90-90-90 targets [7] is to drastically reduce incidence produced by long transmission chains. In other words, the aim is for 90% of all people living with HIV to be diagnosed, 90% of diagnosed individuals to receive antiretroviral therapy, and 90% of treated individuals to have viral suppression. Also, quantifying the role of early transmission in the epidemic is important from a public health standpoint, as it can help assess the extent to which programs are able to reach infected individuals early enough. This is the motivation behind the current study, in which we analyse a large sample of HIV sequences collected via the Quebec HIV genotyping program with a variety of methods, comparing estimates of growth in transmission chains to shed light on their sensitivity to modelling assumptions, thus better framing the recent transmission dynamics of the epidemic.

There is a lack of consensus as to how to best partition HIV-1 sequence data into distinct transmission chains, and different methods may produce equally valid, but conflicting results [8]. This paper therefore compares several up-to-date estimates of transmission chains in the HIV epidemic among MSMs in Quebec and looks into their temporal expansion, improving as a result previous assessments of the contribution of early transmission to the epidemic. More specifically, we consider the following methods:

1. Maximum likelihood phylogenetic reconstruction coupled with several combinations of bootstrap support and genetic distance requirements for clades, termed the *ML-bootstrap* approach,
2. Bayesian phylogenetic inference, coupled with several combinations of posterior probability support and genetic distance requirement for clades,
3. DM-PhyClus [9], a Bayesian method that defines chains as sets of sequences supported by subtrees with distinctive branch length distributions,
4. The Gap Procedure [10], a non-phylogenetic distance-based approach that requires chains to be reasonably distinctive from one another.

## Materials and methods

### Sequence database

The Quebec HIV genotyping program database, operational since 2002, includes HIV pol sequences from all genotyped persons at the Jewish General Hospital (site supervisor: Bluma Brenner) and Hôpital Notre-Dame (site supervisor: Michel Roger) genotyping sites. All blood samples, sequences, and medical records at the two testing sites were fully anonymized and encrypted before analysis. A unique phylogenetic identifier number was assigned for each person. It was created based on putative cluster group association, using a birthdate cross-linker to rule out repeat patient sampling. First genotypes were used in those analyses. The database can still include multiple sequences for certain patients, in which case letters are appended to the identifier to indicate collection order, e.g. C001-001a, C001-001b. The putative clusters were subjected to BLAST, to ensure that transmissions were local.

### Provincial cohort of newly-infected MSMs (2002-2016)

Among the entries in the Quebec HIV genotyping program database [11], we first retain 3,916 subtype B sequences, each obtained from a different individual classified as MSM. Sequences include 918 sites located in the *pol* gene: positions 10-297 of the protease region (PR), and 112-741 of the reverse transcriptase (RT) region. All sequences were collected between May 3rd, 1996 and February 1st, 2016. When multiple sequences were available for a single individual, we selected the one with the earliest sampling date. Sequences come with a time stamp, indicating when the blood sample was collected, and an indicator of infection status, either chronic treated, chronic untreated, or Primary HIV Infection (PHI). A case is considered a PHI if the sequence was obtained less than six months after seroconversion [11]. A physician was responsible for the assessment of PHI status, based on serologic testing algorithms for recent HIV infection [12].

Genotypic drug resistance testing is standard of care for all persons diagnosed with HIV, and test requisition forms identify all newly-diagnosed or treatment-naïve persons. All newly-diagnosed persons are genotyped prior to treatment onset and so, do not have acquired resistance. The database included 212 sequences from chronic treated persons or persons with missing infection status, which we exclude to prevent artefacts in clustering due to selective pressure induced by treatment. Transmitted resistance on the other hand is limited to only a few single point mutations, K103N and G190A for example. Past analyses of a previous version of the database [11, 13] have revealed that chain membership estimation was not affected by their inclusion. We are left with 3,704 sequences, of which 1,401 were sampled from individuals in the PHI stage. Finally, for rooting purposes, we add to those 3,704 sequences three subtype A outgroup sequences from Zambia [14] (NCBI accession numbers AB254141, AB254142, AB254143), and it follows that the dataset we consider includes 3,707 sequences in total. Subtype A sequences are sufficiently different from subtype B sequences to make appropriate outgroups. As well as allowing root placement, they serve as a crude check of inference quality. Indeed, any phylogeny in which subtype A sequences are placed inside subtype B transmission chains would be discarded outright.

### Chain estimation

We present a brief review of phylogenetic concepts in Supplementary Material S1. We obtain the maximum likelihood (ML) phylogenetic estimate [15] with RAxML 8.2.10 [16], under the assumption that nucleotide evolution follows the GTR + I + Γ(5) model. We produce 1,000 bootstrap trees, and use them to evaluate confidence in the clades found in the estimated ML phylogeny. To obtain estimates of transmission chains, we separately consider 15 combinations of bootstrap support and genetic distance requirements. More specifically, we first apply a bootstrap support requirement of either 70%, 90%, or 95%. We then consider a distance requirement of either 1.5%, 3.0%, 4.5%, or one corresponding to the 15th or 30th percentile of the maximum likelihood tree’s patristic distance distribution [17]. We apply the genetic distance criterion in one of three ways: we use, in turns, the maximum within-chain *p*-distance requirement of ClusterPicker [18], the maximum median within-chain patristic distance requirement of PhyloPart [17], and the maximum within-chain patristic distance requirement found in [11]. For the PhyloPart analysis, we consider a combination of the bootstrap requirements listed before, but, per guidelines in [17], only the two distance cutpoints based on percentiles of patristic distances.

We also perform phylogenetic inference with MrBayes 3.2.6 [19] using default parameters, under the assumption that nucleotide evolution follows the GTR + I + Γ(4) model. MrBayes uses the Markov Chain Monte Carlo (MCMC) algorithm [20], more specifically the so-called *Metropolis-coupled MCMC*, or (MC)^3^[21], algorithm, to generate estimates for the posterior distribution of phylogenetic parameters. The MCMC algorithm lets us obtain samples from the posterior distributions of interest. It starts off by setting all parameters at an arbitrary value. Then, in each iteration, updates to parameter values are proposed, conditional on their current values. Each proposal is randomly accepted with probability equal to the Metropolis-Hastings (MH) ratio, producing a move in the parameter space; else, no move is recorded. After a large number of iterations, parameter values generated throughout the chain are used to empirically estimate the posteriors. We run three million iterations, burning in the first 50% and sampling one iteration out of 500. We derive the majority rule consensus tree from the remaining 3,000 trees, and produce chain estimates by identifying clades with posterior probability support of 1.0. We picked this requirement because it is, by far, the most commonly encountered in the HIV clustering literature, e.g. [22–25]. Studies that use posterior probability requirements under 1.0 are fairly rare [26, 27]. Since the majority rule consensus tree does not have branch lengths, we use the ClusterPicker algorithm to obtain chain estimates, under all distance requirements stated earlier. We therefore produce five additional partitions.

DM-PhyClus is a Bayesian phylogenetic algorithm that estimates transmission chains by identifying disjoint sets of sequences supported by subtrees with distinctive branch length distributions. It therefore avoids patristic distance and clade support requirements [9]. Conditional on an input phylogeny—the maximum likelihood estimate in this study—it uses MCMC to produce an estimate of the posterior distribution of chain membership indices. As a result, it also provides a straightforward measure of uncertainty for chain membership indices, in the form of co-clustering frequencies across the MCMC run. It requires specification of a number of other priors and evolutionary parameters, which we list in Supplementary Material S4. We perform 220,000 iterations, discarding the first 20,000 as a burn-in and applying a thinning ratio of 1 over 200, leaving us with a sample of size 1,000. We identify the partition that maximises the joint posterior probability score, which we refer to as the Maximum Posterior probability (MAP) estimate. We also derived the so-called *linkage estimate* from the MCMC run results [9]. To obtain the linkage estimate, we first derive an *adjacency matrix* from each partition visited in the mcmc run. The adjacency matrix is a square matrix with entry (*i*, *j*) taking value 1 if sequences *i* and *j* are in the same transmission chain, and 0 otherwise. We then compute a mean of all the obtained matrices, and round up to 1 all elements above a pre-specified threshold, e.g. 0.7, and down to 0 all the others. We consider the resulting matrix a representation of an undirected unweighted network. The distinct components, called *modules*, it contains form the linkage estimate. We present a more detailed description in Supplementary Material S5.

The Gap Procedure is a pure distance-based partitioning algorithm that avoids reliance on ad hoc cutpoints by separating sets of sequences into distinctive components without requiring phylogenetic estimation [10]. When the true chains are compact and separable enough, the Gap Procedure can propose partitions that largely agree with conventional phylogenetic estimates, but in a fraction of the time normally required for such analyses, thus making the method ideal for handling large datasets. For example, in an analysis presented in [10], partitioning a dataset comprising 627 sequences of length 810 took 126 hours with MrBayes and less than a second with the Gap Procedure. The method takes as sole input a matrix of *p*-distances, which we obtain under the Kimura 1980 (K80) model. We leave tuning parameters at their default values.

### Cutpoint selection for the conventional maximum likelihood and Bayesian methods

We wish to retain only one chain estimate per method used, and must therefore select a “best” partition among those proposed. Prior to the analyses, clinicians involved in the Quebec HIV genotyping program had performed a preliminary partitioning of the dataset. The partition they proposed results from successive updates, performed at different points in time, of an initial set of transmission chains. They inferred the initial set by imposing very stringent bootstrap support (≥ 98%) and maximum within-chain patristic distance requirements (< 0.015 nt/bp) [11]. Such conservative thresholds are meant to ensure that any two sequences found to co-cluster indeed belong to the same transmission chain. In the following updates, once a sequence is attributed to a chain, it cannot be re-attributed to another chain. They identified from the results seven noteworthy chains comprising 372 sequences in total, which we use as a reference [17]. One of those chains, for instance, has 68 sequences, among which more than half harbour the Non-Nucleoside Reverse Transcriptase Inhibitors (NNRTI) mutation K103N. We compare all partitions we obtain with that reference using the Adjusted Rand Index (ARI), a measure of similarity between two partitions, with the aim of maximising overlap. Greater ARI values are better, and the measure is bounded above by 1, indicating perfect correspondence. We describe the comparison scheme in more details in Supplementary Material S2.

### Growth of transmission chains

To evaluate the growth of transmission chains, ideally, we would need to know seroconversion dates for all cases whose sequences were sampled. Indeed, it is not enough to infer that a sequence belongs to a known chain: it might be that the infection event happened a long time ago, but was only diagnosed recently. In that situation, the chain did not actually expand: we are merely observing the effects of an increase in the sampling rate. This is a common problem in transmission chain inference [6]. The dataset contains an infection stage indicator, equivalent to a censored estimate of infection time, i.e. smaller (greater or equal) than six months prior to the sampling date for PHIs (chronic cases). Since HIV genetic diversity is low in newly-infected individuals [28], we can more accurately associate PHIs to the different chains. Most HIV-positive individuals are diagnosed while already in the chronic stage, at which point seroconversion date estimates are very imprecise [28]. As a result, we decide to use PHIs only to obtain a lower bound estimate for the growth of inferred chains, since infection dates for PHIs can be placed reliably within a short time window prior to sampling. We focus on a period ranging from January 1, 2012 to February 1, 2016. For example, one of the methods may propose a chain of size 20, with eight of its sequences having been obtained from cases diagnosed while in the PHI stage at some point in 2014. We can therefore be certain that those cases were infected after January 1, 2012 and so, we conclude that the chain has accrued at least eight new cases in the selected period.

### Software

We import phylogenetic estimates from RAxML or MrBayes into R v3.2.3 and produce partitions of the data with custom functions built with the help of the *phangorn* and *ape* libraries [29]. We use functions in the *GapProcedure* and *DMphyClus* R libraries to obtain the other estimates.

## Results

### Cutpoint selection

The 15th and 30th percentiles of the maximum likelihood tree’s patristic distance distribution are equal to 6.8% and 7.7%, respectively. In all maximum likelihood analyses, the bootstrap support requirement of 70% resulted in greater overlap with the reference. Under the maximum patristic distance scheme of [11], we found that a distance requirement of 7.7% maximised the correspondence (ARI = 0.91). With ClusterPicker, requirements of either 6.8% or 7.7% were preferable (ARI = 0.91). In PhyloPart, a median within-chain patristic distance requirement of 0.03 resulted in the largest overlap with the reference (ARI = 0.98). Finally, in the Bayesian analysis, in addition to a posterior probability requirement of 1, we determined that a 6.8% or 7.7% requirement for maximum within-chain *p*-distances were equivalent (ARI = 0.91). In cases where several distance requirements were equivalent, we picked the smallest one.

### Estimates comparison

We first compare optimal partitions from all methods with the ARI, cf. Table 1. As expected, the DM-PhyClus linkage and MAP estimates are very similar (ARI = 0.99). Besides those, we observed the largest overlap between the partitions resulting from the *ML + ClusterPicker* and *MrBayes + ClusterPicker* methods (ARI = 0.94). DM-PhyClus produced the most distinctive partition, producing overlap with other methods ranging from 0.64 and 0.72. The larger correspondence with the Gap Procedure estimate is not surprising, since both methods look for chains that are well separated.

**Table 1:** Adjusted Rand index for the overlap between the chain estimates obtained from the different methods. CP stands for ClusterPicker.

	ML + CP	ML + PhyloPart	ML + Max. pat. dist.	MrBayes + CP	Gap Procedure	DM-PhyClus + MAP	DM-PhyClus + Linkage
ML + CP	1.00	0.92	0.93	0.94	0.84	0.65	0.64
ML + PhyloPart	0.92	1.00	0.91	0.86	0.90	0.68	0.68
ML + Max. pat. dist.	0.93	0.91	1.00	0.88	0.85	0.66	0.66
MrBayes + CP	0.94	0.86	0.88	1.00	0.84	0.64	0.64
Gap Procedure	0.84	0.90	0.85	0.84	1.00	0.72	0.72
DM-PhyClus + MAP	0.65	0.68	0.66	0.64	0.72	1.00	0.99
DM-PhyClus + Linkage	0.64	0.68	0.66	0.64	0.72	0.99	1.00

**Fig 1:**
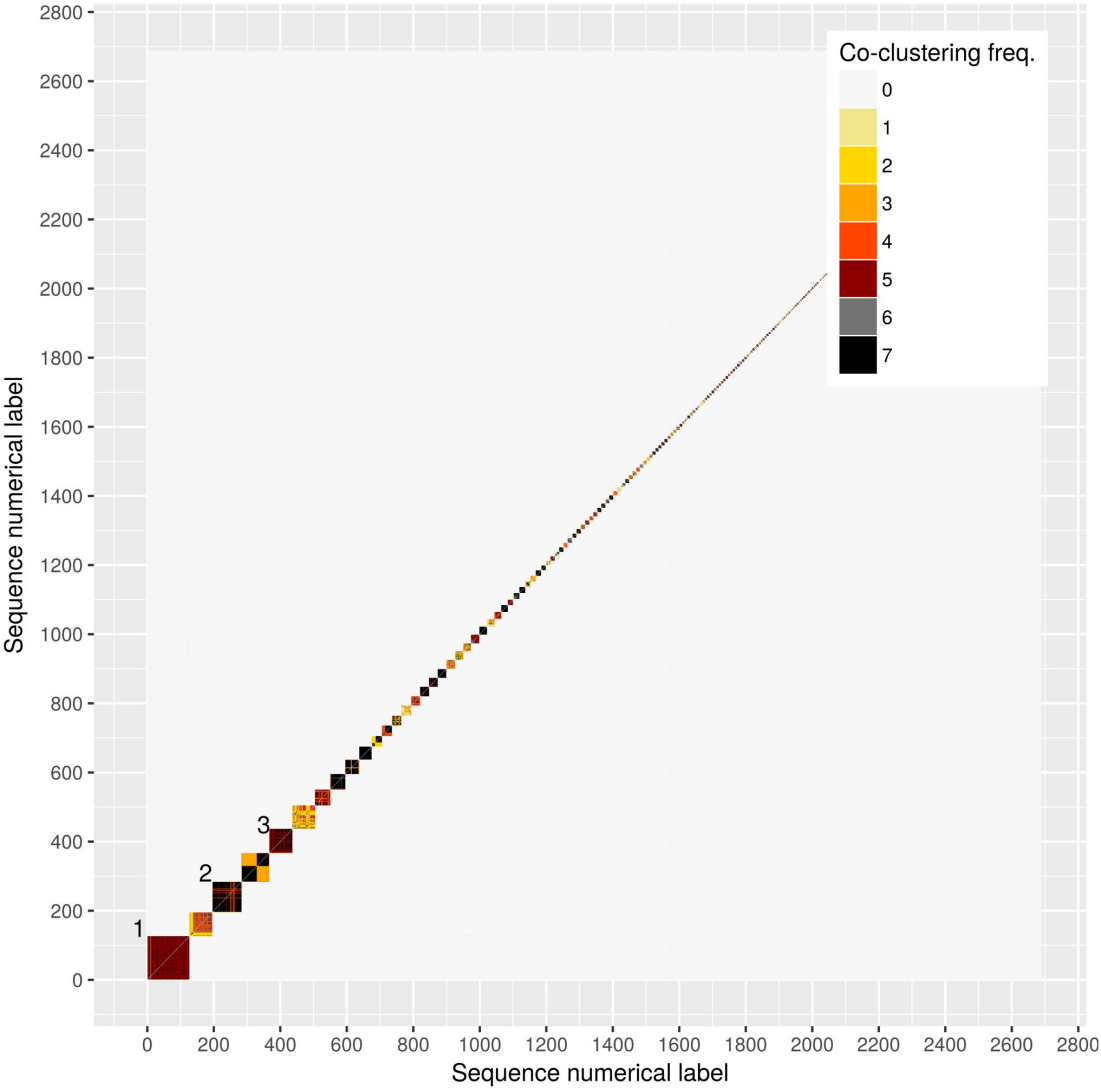
Heat map indicating overlap between estimates produced by the seven methods considered. We excluded all sequences that were found to be singletons by the seven methods we used, leaving us with 2,938 sequences. We gave each remaining sequence a numerical label between 1 and 2,938. The colour of each pixel indicates the number of methods for which the two sequences identified by the *x* and *y* axes co-clustered.

We depict in Fig 1 the correspondence between the seven partitions we computed. Each square in the heat map is made up of a large number of pixels, each pixel indicating the number of methods that produced partitions attributing to the same chain the sequences labelled by the *x* and *y* axes. In other words, for any two distinct sequences, we counted how many of the seven estimates had them in the same chain: this is what the graph represents. We only show the 2,938 sequences found to co-cluster with at least one other sequence by at least one of the methods. The graph reveals several moderately-sized chains. The largest rectangle, marked “1” in the figure, includes 126 sequences, 124 of which belonged to the same reference chain. The earliest sequence in the block, a PHI, was collected on August 13th, 2002, and the latest, also a PHI, on December 23rd, 2015, which suggests the chain might still be expanding. The MAP and linkage estimates of DM-PhyClus, on the other hand, split this block into 14 components, including three chains of size 37, 36, and 14, respectively, and 7 singletons.

The second largest block, marked “2” in the figure, comprises 88 sequences, 87 of which co-clustered in the reference set. Its sequences were sampled between May 11st, 2004 (chronic untreated) and December 14, 2015 (PHI), which highlights its durability. Of its 3,828 pairs of sequences, 2,932 were found to be in the same chain by all methods, and 395, by five of the seven methods. The block marked “3” also stands out. Among its 71 sequences, 68 were found in the same reference chain. Its first sequence (PHI) was collected on August 13th, 2002 and its last (chronic untreated), on March 28th, 2015. Of its 2,415 unique pairs of sequences, 1,066 were found to co-cluster by all methods, and 1,280, by five of the seven methods.

Table 2 provides summary statistics for the inferred chains. The four conventional methods produced chain length distributions with similar characteristics. We found that ML + PhyloPart and DM-PhyClus + Linkage had the highest proportions of singletons, with approximately 36% of sequences not co-clustering with any other sequence. Further, DM-PhyClus did not identify the size 126 transmission chain found by the other methods, as we had noted when we commented on block 1 in Fig 1. On the other hand, the Gap Procedure and the conventional Bayesian estimate had the fewest singletons. The Gap Procedure estimate, however, contained many more transmission pairs.

**Table 2:** Summary statistics for estimates returned by the different methods.

	ML + ClusterPicker	ML + PhyloPart	ML + Max. pat. dist.	MrBayes + ClusterPicker	Gap Procedure	DM-PhyClus + MAP	DM-PhyClus + Linkage
Mean chain length	2.29	2.08	2.19	2.48	2.38	2.11	2.08
Mean (no singletons)	6.01	5.53	5.62	5.96	4.46	5.62	5.65
Median (no singletons)	3.00	3.00	3.00	3.00	2.00	3.00	3.00
Max. chain length	126	126	126	126	125	77	77
Num. singletons	1205	1353	1261	1051	937	1330	1373
Num. chains ≥ 2	1621	1779	1696	1497	1558	1753	1786

### Transmission chain growth assessment

Among the 957 cases in the MSM risk group added to the database in the period ranging from January 1st, 2012 to February 1st, 2016, 304 were PHIs. Of those PHIs, 254 were sampled after June 30th 2012, guaranteeing that the corresponding transmission events took place in 2012. According to the *ML + PhyloPart* estimate, 50 (20%) of those 254 PHIs are singletons, 23 (9%) are found in transmission pairs, and 153 (60%) belong to chains of length five or more. In comparison, in the period ranging from July 1st 2008 to January 1st 2012, 319 MSM cases diagnosed in the PHI stage were added to the database. Out of those, 83 (26%) are singletons, 34 (11%) belong to transmission pairs, and 159 (50%) are part of chains of length five or more.

**Fig 2:**
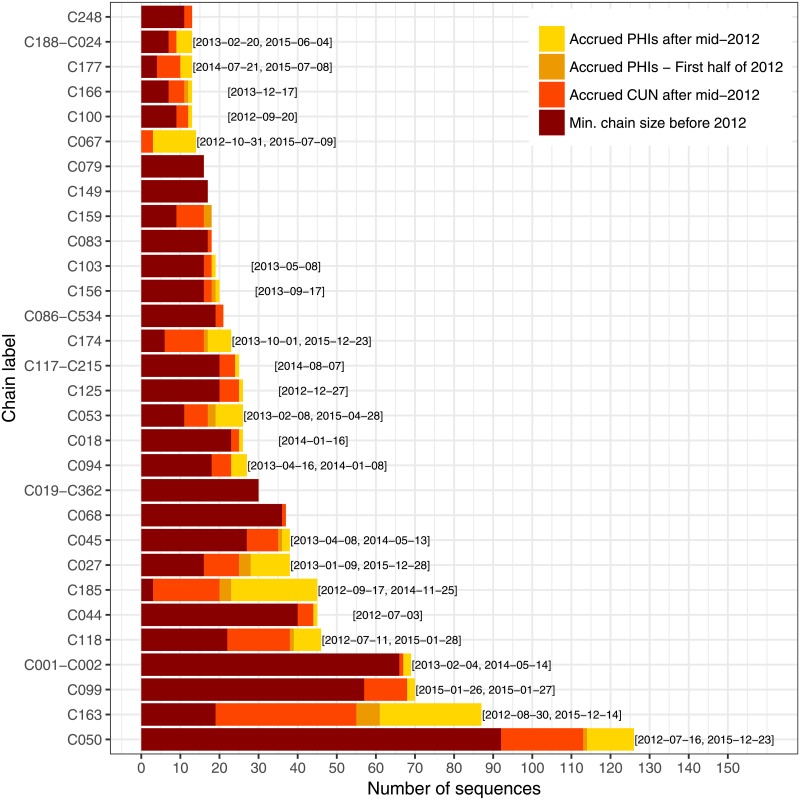
Bar plot showing the breakdown in membership for the 30 longest chains in the ML + PhyloPart estimate. The labels at the end of each bar indicate the sequence collection dates for the first and last “recent” PHIs in the chain, that is, recorded on or after July 1st, 2012. When there is only one such PHI, we display the corresponding collection date instead. We assume that all chronic cases recorded before July 1st, 2012 were infected prior to 2012. The dark red bar represents the “minimum chain size before 2012” because several chronic cases diagnosed after July 1st 2012 were probably infected prior to 2012. Also, it is likely that several PHIs sampled in the first half of 2012 match with transmission events that occurred late in 2011.

**Fig 3:**
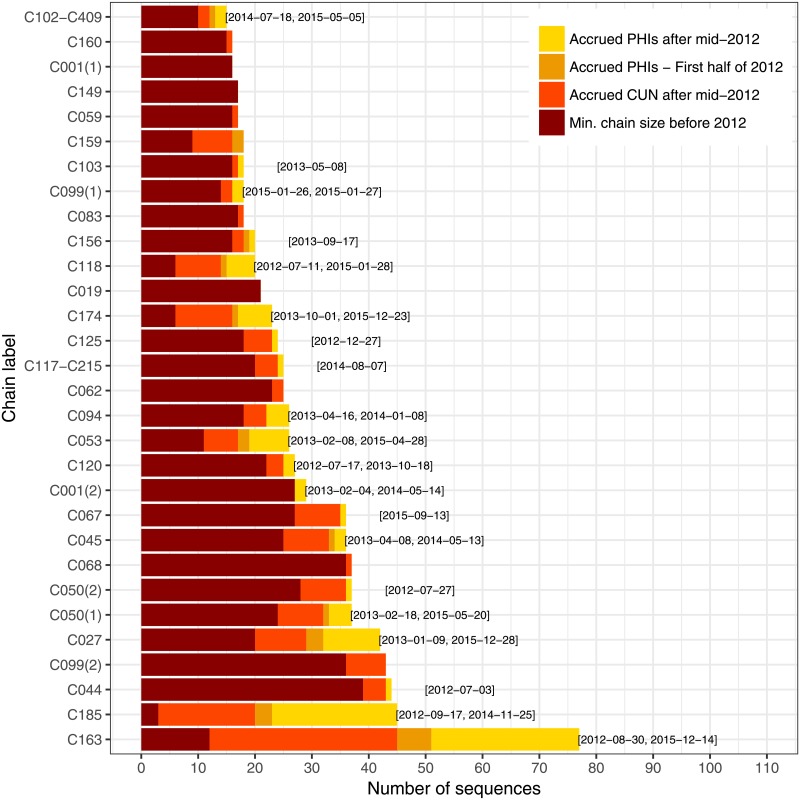
Bar plot showing the breakdown in membership for the 30 largest chains in the DM-PhyClus + MAP estimate. The labels at the end of each bar indicate the sequence collection dates for the first and last “recent” PHIs in the chain, that is, recorded on or after July 1st, 2012. When there is only one such PHI, we display the corresponding collection date instead. We assume that all chronic cases recorded before July 1st, 2012 were infected prior to 2012. The dark red bar represents the “minimum chain size before 2012” because several chronic cases diagnosed after July 1st 2012 were probably infected prior to 2012. Also, it is likely that several PHIs sampled in the first half of 2012 match with transmission events that occurred late in 2011.

We represent the 30 longest chains, according to the *ML + PhyloPart* estimate, in Fig 2. The labels on the *y* axis are based on transmission chains described in [3, 30]. When we found a chain with members from more than one such chain, we created a new label by concatenating the numbers of the two largest chains, under the condition that the second largest was not a singleton. Those 30 chains include 126 recent PHIs, split between 22 chains. Among the ten longest chains, nine include at least one recent PHI. The longest chain includes 12 recent PHIs, while the second and third include 26 and two, respectively. Chain C185 is noteworthy: despite its smaller length prior to 2012, it has grown quickly, with the addition of 22 recent PHI. Chain C067, on the other hand, is still short, but had not been recorded before 2012. Each of those two chains has a PHI recorded as late as the second half of 2015, indicating that they may still be expanding. The partition produced by DM-PhyClus + MAP is different, but leads to similar conclusions, as shown in Fig 3. Of the 30 longest chains, 20 include at least one recent PHI. We note that the longest chain includes sequences from transmission chain C163; other methods found instead that the longest chain comprised sequences from chain C050, which DM-PhyClus split up. Other conventional estimates, the Gap Procedure, and DM-PhyClus + Linkage lead to similar conclusions, as can be seen in Supplementary Material S6. The split of C099 in the DM-PhyClus estimate is also meaningful, as it reflects successive waves of infections. All sequences in C099(01) are from a wild-type virus. In C099(2), 36 out of 43 sequences harbour transmitted drug-resistance mutation K103N, four out of 43 harbour mutation V106I, and finally, only three out of 43 are from a wild-type virus.

## Discussion

Phylogenetic analyses can help in the prioritization of public health interventions by highlighting high-risk transmission groups. Previous findings had suggested that long chains play a substantial role in sustaining HIV epidemics in the era of Treatment-as-Prevention [3]. In Quebec, people belonging to long chains are mostly MSMs, younger, and concentrated in the greater Montreal area [3]. Viral species found in long chains may be characterised by improved replicative fitness and the ability to overcome transmission barriers [31]. Moreover, poor testing habits may exacerbate the persistence of long chains [3]. It remains important to identify and target growing transmission chains in near real-time [32, 33].

This study compared several phylogenetic approaches applied to a subset of a large HIV sequence database, representative of the molecular diversity in the HIV epidemic among MSMs in Quebec. More specifically, we looked at HIV-1 transmission chains among 3,704 HIV-1 cases belonging to the MSM risk category. We compared estimates from seven methods, four conventional approaches relying on a variety of cutpoints applied to phylogenetic estimates, and three additional recent approaches seeking to reduce reliance on cutpoints, the Gap Procedure and two variations on DM-PhyClus. We found that estimates obtained from conventional methods were overall fairly similar. The estimates from DM-PhyClus involved a noticeably different chain length distribution. All estimates however produced a similar assessment of chain growth in the period ranging from January 1st, 2012 to February 1st, 2016: nine of the ten longest chains had grown in the selected period, three of those having accrued at least ten new cases. Further, we observed emerging chains, as well as an increase in the proportion of recent PHIs belonging to chains of size 5 or more.

The study has several limitations. Cutpoint selection remains inherently subjective. In the current work, the observed similarities in the proposed choices of cutpoints were not surprising. Chain estimation based on the consensus tree obtained after running the MCMC algorithm relied on ClusterPicker, just like one of the ML-bootstrap approaches. Often, chains found through a Bayesian analysis agree substantially with those obtained through a sensibly-tuned ML-bootstrap approach [22, 27]. Also, ClusterPicker uses maximum within-chain *p*-distances, which provide a rough approximation of patristic distances. It follows that tuning ClusterPicker to produce estimates in line with those from the method of [11] is possible.

Choosing cutpoints as to maximise overlap with a reference set does not guarantee that other chains will be estimated well. Moreover, identifying a suitable reference set can be difficult. In our study, researchers involved in the Quebec HIV genotyping program proposed the set. A different reference set might have led to different cutpoints. Several of the approaches we used did manage to recover the reference set very closely though, which suggests that it is not unrealistic.

DM-PhyClus, being a Bayesian method, rests on a number of prior assumptions, which are all more or less informative, and it follows that prior calibration is key. Although estimates are reasonably robust to some prior assumptions [9], it remains possible that a combination of very poorly chosen priors may result in misleading chain estimates. Further, DM-PhyClus looks for branch length patterns hinting at the existence of transmission cascades. This study focused instead on transmission chains, which do not necessarily involve such patterns. Therefore, the assumptions underlying DM-PhyClus might not be entirely appropriate.

Reliable infection date estimates for cases diagnosed while in the chronic stage are unavailable and so, we could only obtain a lower bound for chain growth between January 1st, 2012 and February 1st, 2016. Time between seroconversion and diagnosis can go from months to years, depending on an individual’s testing habits, with a mean that could be above two years [34–36], and it follows that several chronic cases diagnosed after June 30th, 2012 might have been infected during the selected period. Estimating infection time from the fraction of ambiguous nucleotides in each sequence would have been possible [28], but the high standard deviation for such predictions would have limited their usefulness.

Because of the non-random sampling of cases, we cannot readily deduce from our estimates the population-level chain length distribution. In the absence of covariate information, we cannot model the sampling process. If, for example, the probability for a case to be sampled correlates positively with chain length, we might end up underestimating the length of shorter chains and the number of singletons, and consequently, overestimating the contribution of the chains we found to the epidemic. Nevertheless, the results we presented provide good evidence of chain growth, and that phenomenon alone warrants attention.

Determining which partition among the six proposed provides the most accurate representation of transmission chains in the sample is difficult. The choice depends ultimately on our confidence in the assumptions of each approach, and on substantive knowledge. The agreement between estimates from the conventional approaches, although explained in great part by shared assumptions, is still a good sign. The moderately different partitions proposed by the Gap Procedure and DM-PhyClus are not erroneous: they result from the way the two methods define a partition of interest. The two approaches also have additional aims and benefits. [10] designed the Gap Procedure with scalability in mind, and [9] formulated DM-PhyClus in such a way that it could offer a straightforward measure of uncertainty for its estimates.

The existence of long transmission chains is not only a feature of transmission of HIV-1 among MSMs in Montreal: it has been observed across Europe and other regions of North America as well [22, 37–39]. The increasing size of sequence databases represents a considerable computational challenge, especially in the Bayesian framework, and so, scalability should be an essential feature of future partitioning algorithms. We contend that methods that avoid cutpoint selection altogether are promising, and would benefit from further improvements. In addition to lightening their computational burden, adapting them to use time-stamp and covariate data, for example, would be a welcome extension. Further, methods designed to provide a clear measure of uncertainty for estimated partitions, like DM-PhyClus, would warrant more attention. Indeed, the strength of co-clustering between sequences within an inferred chain may vary sizeably, and the separation between some chains may not be very clear-cut. Such variability may be hard to measure rigorously under conventional phylogenetic partitioning approaches.

Phylogenetic surveillance of HIV transmission among MSMs provides helpful clues for explaining the persistence of the epidemic. Results from this study should help inform public health strategies to reduce transmission rates.

## Ethics statement

The current study did not involve data collection, as it is a secondary analysis of viral sequence data collected for the Quebec HIV genotyping program. It has received ethics approval from the McGill Faculty of Medicine Institutional Review Board (A08-M25-17B, approved on August 28, 2017).

Baseline genotyping is routinely performed in all HIV-infected participants without informed written consent. Ethics approval for encrypted and non-nominative phylogenetic surveillance of the HIV epidemic was obtained from the Ministère de la Santé and L’Institut national de santé publique du Québec in 2008. Continuing annual ethics approval (CODIM-MBM-15-185, renewed January 26, 2018) was obtained from the Centre Intégré universitaire de santé et de service sociaux du Centre-Ouest-de-l’Île-de-Montréal—Hôpital général juif, in accordance with the Declaration of Helsinki, national and institutional standards.

## Supporting information

S1 Text A review of phylogenetic concepts. (PDF)

S2 Text Cutpoint selection with a partial gold standard. (PDF)

S3 Text MrBayes script. (PDF)

S4 Text Tuning parameters used in the DM-PhyClus and Gap Procedure analyses. (PDF)

S5 Text The linkage estimate. (PDF)

S6 Text Additional bar plots depicting chain growth between January 1st, 2012 and February 1st, 2016. (PDF)

## References

[1] Summary: Estimates of HIV incidence, prevalence and proportion undiagnosed in Canada, 2014; 2015. Available from: http://healthycanadians.gc.ca/publications/diseases-conditions-maladies-affections/hiv-aids-estimates-2014-vih-sida-estimations/index-eng.php#t2.

[2] Public Health Agency of Canada. Population-specific HIV/AIDS Status Report: Gay, Bisexual, Two-Spirit And Other Men Who Have Sex With Men; 2013. Available from: http://www.phac-aspc.gc.ca/aids-sida/publication/ps-pd/men-hommes/chapter-chapitre-3-eng.php.

[3] Brenner BG, Ibanescu RI, Hardy I, Stephens D, Otis J, Moodie E, et al Large cluster outbreaks sustain the HIV epidemic among MSM in Quebec. AIDS. 2017;31(5):707–717. 10.1097/QAD.0000000000001383 28005684

[4] Charest H, Doualla-Bell F, Cantin R, Murphy DG, Lemieux L, Brenner B, et al A Significant Reduction in the Frequency of HIV-1 Drug Resistance in Québec from 2001 to 2011 Is Associated with a Decrease in the Monitored Viral Load. PLOS ONE. 2014;9(10):1–10. 10.1371/journal.pone.0109420 PMC4190276 25295725

[5] Brenner B, Wainberg MA, Roger M. Phylogenetic inferences on HIV-1 transmission: implications for the design of prevention and treatment interventions. AIDS. 2013;27(7):1045–1057. 10.1097/QAD.0b013e32835cffd9 23902920 PMC3786580

[6] Poon AF. Impacts and shortcomings of genetic clustering methods for infectious disease outbreaks. Virus Evol. 2016;2(2):vew031 10.1093/ve/vew031 28058111 PMC5210024

[7] 90-90-90—An ambitious treatment target to help end the AIDS epidemic; 2017. Available from: http://www.unaids.org/sites/default/files/media_asset/90-90-90_en.pdf.

[8] Brenner BG, Wainberg MA. Future of Phylogeny in HIV Prevention. J Acquir Immune Defic Syndr. 2013;63 Suppl 2:S248–S254. 10.1097/QAI.0b013e3182986f96 23764643 PMC3689150

[9] Villandre L, Labbe A, Brenner B, Roger M, Stephens DA. DM-PhyClus: A Bayesian phylogenetic algorithm for transmission cluster inference. ArXiV. 2017;. 10.1186/s12859-018-2347-3 PMC6137936 30217139

[10] Vrbik I, Stephens DA, Roger M, Brenner BG. The Gap Procedure: for the identification of phylogenetic clusters in HIV-1 sequence data. BMC Bioinformatics. 2015;16:355 10.1186/s12859-015-0791-x 26538192 PMC4634160

[11] Brenner BG, Roger M, Routy JP, Moisi D, Ntemgwa M, Matte C, et al High rates of forward transmission events after acute/early HIV-1 infection. J Infect Dis. 2007;195(7):951–959. 10.1086/512088 17330784

[12] Routy JP, Machouf N, Edwardes MD, Brenner BG, Thomas R, Trottier B, et al Factors associated with a decrease in the prevalence of drug resistance in newly HIV-1 infected individuals in Montreal. AIDS. 2004;18(17):2305–2312. 10.1097/00002030-200411190-00011 15577543

[13] Brenner BG, Roger M, Moisi DD, Oliveira M, Hardy I, Turgel R, et al Transmission networks of drug resistance acquired in primary/early stage HIV infection. AIDS. 2008;22(18):2509–2515. 10.1097/QAD.0b013e3283121c90 19005274 PMC2650396

[14] Los Alamos HIV sequence database;. Available from: http://www.hiv.lanl.gov.

[15] Felsenstein J. Evolutionary trees from DNA sequences: A maximum likelihood approach. J Mol Evol. 1981;17(6):368–376. 10.1007/BF01734359 7288891

[16] Stamatakis A. RAxML Version 8: A tool for Phylogenetic Analysis and Post-Analysis of Large Phylogenies. Bioinformatics. 2014; 10.1093/bioinformatics/btu033 PMC3998144 24451623

[17] Prosperi MCF, Ciccozzi M, Fanti I, Saladini F, Pecorari M, Borghi V, et al A novel methodology for large-scale phylogeny partition. Nat Commun. 2011;2:321 10.1038/ncomms1325 21610724 PMC6045912

[18] Ragonnet-Cronin M, Hodcroft E, Hué S, Fearnhill E, Delpech V, Brown AJL, et al Automated analysis of phylogenetic clusters. BMC Bioinformatics. 2013;14:317 10.1186/1471-2105-14-317 24191891 PMC4228337

[19] Ronquist F, Teslenko M, van der Mark P, Ayres DL, Darling A, Höhna S, et al MrBayes 3.2: Efficient Bayesian Phylogenetic Inference and Model Choice Across a Large Model Space. Syst Biol. 2012;61(3):539–542. 10.1093/sysbio/sys029 22357727 PMC3329765

[20] Hastings WK. Monte Carlo Sampling Methods Using Markov Chains and Their Applications. Biometrika. 1970;57(1):97–109. 10.1093/biomet/57.1.97

[21] Geyer CJ. Markov chain Monte Carlo maximum likelihood. In: Karamigas E, Kaufman S, editors. Proceedings of 23rd Symposium on the Interface. Fairfax, Virginia: Interface Foundation of North America; 1992. p. 156–163.

[22] Lewis F, Hughes GJ, Rambaut A, Pozniak A, Brown AJL. Episodic sexual transmission of HIV revealed by molecular phylodynamics. PLoS Med. 2008;5(3):e50 10.1371/journal.pmed.0050050 18351795 PMC2267814

[23] Chalmet K, Staelens D, Blot S, Dinakis S, Pelgrom J, Plum J, et al Epidemiological study of phylogenetic transmission clusters in a local HIV-1 epidemic reveals distinct differences between subtype B and non-B infections. BMC Infect Dis. 2010;10:262 10.1186/1471-2334-10-262 20822507 PMC2940905

[24] Callegaro A, Svicher V, Alteri C, Presti AL, Valenti D, Goglio A, et al Epidemiological network analysis in HIV-1 B infected patients diagnosed in Italy between 2000 and 2008. Infect Genet Evol. 2011;11(3):624–632. 10.1016/j.meegid.2011.01.019 21292031

[25] Zehender G, Ebranati E, Lai A, Santoro MM, Alteri C, Giuliani M, et al Population dynamics of HIV-1 subtype B in a cohort of men-having-sex-with-men in Rome, Italy. J Acquir Immune Defic Syndr. 2010;55(2):156–160. 10.1097/QAI.0b013e3181eb3002 20703157

[26] Yang Z. Computational Molecular Evolution Oxford Series in Ecology and Evolution. Oxford: Oxford University Press; 2006.

[27] Erixon P, Svennblad B, Britton T, Oxelman B. Reliability of Bayesian Posterior Probabilities and Bootstrap Frequencies in Phylogenetics. Syst Biol. 2003;52(5): pp. 665–673. 10.1080/10635150390235485 14530133

[28] Kouyos RD, von Wyl V, Yerly S, Böni J, Rieder P, Joos B, et al Ambiguous nucleotide calls from population-based sequencing of HIV-1 are a marker for viral diversity and the age of infection. Clin Infect Dis. 2011;52(4):532–539. 10.1093/cid/ciq164 21220770 PMC3060900

[29] Schliep KP. phangorn: phylogenetic analysis in R. Bioinformatics. 2011;27(4):592–593. 10.1093/bioinformatics/btq706 21169378 PMC3035803

[30] Brenner BG, Roger M, Stephens DA, Moisi D, Hardy I, Weinberg J, et al Transmission clustering drives the onward spread of the HIV epidemic among men who have sex with men in Quebec. J Infect Dis. 2011;204(7):1115–1119. 10.1093/infdis/jir468 21881127 PMC3164430

[31] Brenner BG, Ibanescu RI, Oliveira M, Roger M, Hardy I, Routy JP, et al HIV-1 strains belonging to large phylogenetic clusters show accelerated escape from integrase inhibitors in cell culture compared with viral isolates from singleton/small clusters. J Antimicrob Chemother. 2017;72(8):2171–2183. 10.1093/jac/dkx118 28472323 PMC7263826

[32] Wertheim JO, Murrell B, Mehta SR, Forgione LA, Kosakovsky PS, Smith DM, et al Growth of HIV-1 Molecular Transmission Clusters in New York City. J Infect Dis. 2018;40:1–11. 10.1093/infdis/jiy431 PMC6217720 30010850

[33] Poon AFY, Gustafson R, Daly P, Zerr L, Demlow SE, Wong J, et al Near real-time monitoring of HIV transmission hotspots from routine HIV genotyping: an implementation case study. Lancet HIV. 2016;3(5):e231—e238. 10.1016/S2352-3018(16)00046-1. 27126490 PMC4853759

[34] van Sighem A, Nakagawa F, De Angelis D, Quinten C, Bezemer D, de Coul EO, et al Estimating HIV incidence, time to diagnosis, and the undiagnosed HIV epidemic using routine surveillance data. Epidemiology. 2015;26(5):653 10.1097/EDE.0000000000000324 26214334 PMC4521901

[35] Otis J, McFadyen A, Haig T, Blais M, Cox J, Brenner B, et al Beyond condoms: risk reduction strategies among gay, bisexual, and other men who have sex with men receiving rapid HIV testing in Montreal, Canada. AIDS Behav. 2016;20(12):2812–2826. 10.1007/s10461-016-1344-7 26961381 PMC5108827

[36] Phillips AN, Cambiano V, Nakagawa F, Brown AE, Lampe F, Rodger A, et al Increased HIV Incidence in Men Who Have Sex with Men Despite High Levels of ART-Induced Viral Suppression: Analysis of an Extensively Documented Epidemic. PLOS One. 2013;8(2):1–8. 10.1371/journal.pone.0055312 PMC3574102 23457467

[37] Leigh Brown AJ, Lycett SJ, Weinert L, Hughes GJ, Fearnhill E, Dunn DT, et al Transmission network parameters estimated from HIV sequences for a nationwide epidemic. J Infect Dis. 2011;204(9):1463–1469. 10.1093/infdis/jir550 21921202 PMC3182313

[38] Bezemer D, van Sighem A, Lukashov VV, van der Hoek L, Back N, Schuurman R, et al Transmission networks of HIV-1 among men having sex with men in the Netherlands. AIDS. 2010;24(2):271–282. 10.1097/QAD.0b013e328333ddee 20010072

[39] Avila D, Keiser O, Egger M, Kouyos R, Böni J, Yerly S, et al Social meets molecular: Combining phylogenetic and latent class analyses to understand HIV-1 transmission in Switzerland. Am J Epidemiol. 2014;179(12):1514–1525. 10.1093/aje/kwu076 24821749

