# Supplementary material for: Assessing the role of transmission chains in the spread of HIV-1 among men who have sex with men in Quebec, Canada
Source: PLoS One. 2019 Mar 6;14(3):e0213366. doi: 10.1371/journal.pone.0213366 (PMC6402664; doi:10.1371/journal.pone.0213366)
Supplement: S1 Text — (PDF) [file pone.0213366.s001.pdf]

Supplementary Material S1: A review of phylogenetic concepts

Phylogenetic models

Phylogenetic models have become a common tool in the study of infectious disease transmission [1–5] and are used to detect transmission chains. Those models use pathogen genetic sequences, collected from infected individuals, to infer the history of the epidemic, which is represented by a tree structure known as a *phylogeny*, e.g. Fig 1. The phylogeny can be fully represented with two components,

1. The *topology*: Represented by a list indicating the order in which the the tips of the tree meet to form internal nodes,

2. The *branch lengths*: Expressed in expected nucleotide substitutions per base pair (nt/bp), they indicate genetic distance.

The first component summarizes *clades* in the tree. In Fig 1, that list would be (with $\{x\}$ designating the viral DNA sequence from patient x): [{1}, ..., {7}, {3, 4}, {6, 7}, {2, 3, 4}, {5, 6, 7}, {1, ..., 4}, {1, ..., 7}, {Outgroup, 1, ..., 7}]. Any set of genetic sequences forms a clade if and only if it contains *all* sequences descended from an arbitrary node. Trivially, all sets of size 1 and the set comprising the entire sample form clades. A set including only viral sequences from patients 1 and 2 would not constitute a clade however, since their most recent common ancestor has four descendants, namely, the sequences from patients 1 to 4.

Due to the availability of sizeable viral DNA sequence databases collected in the context of antiretroviral drug resistance testing [6–8], phylogenetics has been used extensively to study Human Immunodeficiency Virus (HIV) epidemics [9, 10]. The Quebec HIV genotyping program database for example, as of 2017, contains $27,487$ HIV sequences from $9,687$ HIV-positive individuals, living mostly in Montreal, Quebec, Canada [11].

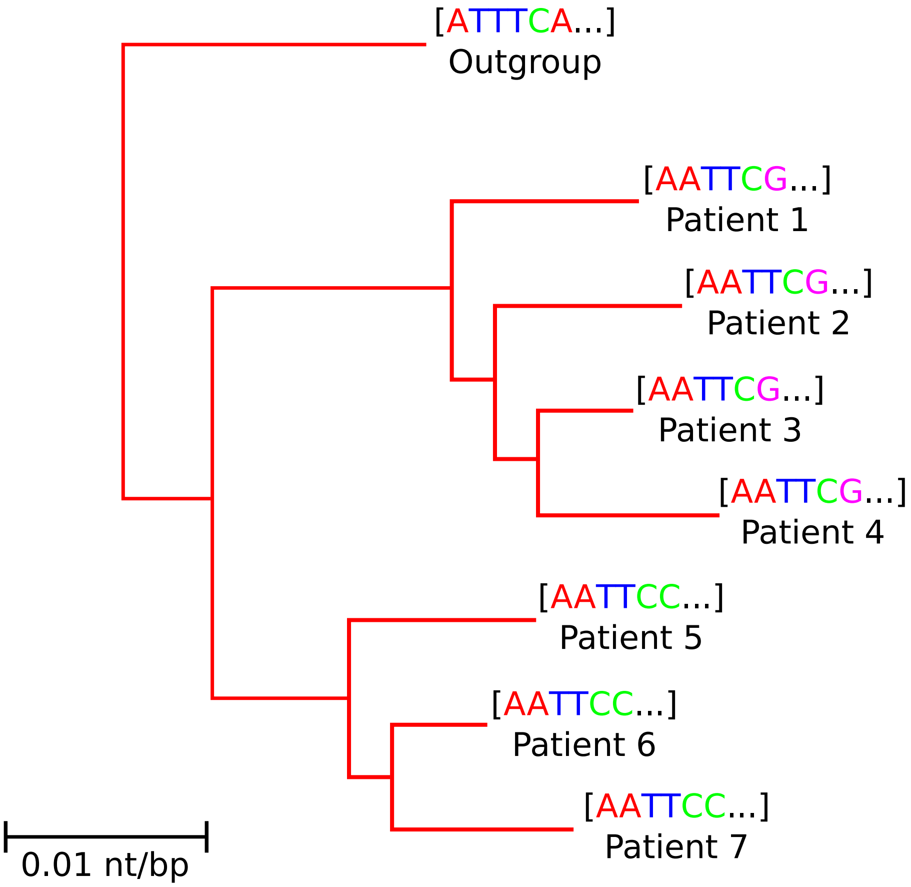

Fig 1. Phylogeny for a sample of seven HIV sequences obtained from different HIV-positive patients, with an outgroup used to place the *root* of the tree. The sampled viral sequences are placed at the tips of the tree, and the root corresponds to the sample's most recent common ancestor. Branches link the different nodes in the tree. Their lengths are expressed in expected nucleotide substitutions per base pair (nt/bp), a measure of genetic distance, indicating dissimilarity between sequences.

Transmission clusters and transmission chains

Disagreements persist in the literature regarding the difference between *transmission chains* and *transmission clusters* [12, 13]. When studying viral transmission among members of a population, we often consider only one viral DNA sequence per infected individual, and therefore, a given clade contains sequences from all sampled individuals who became infected following a specific outbreak or introduction of the pathogen into the population. As a result, those cases must belong to the same transmission chain. All conventional phylogenetic clustering methods require transmission clusters to be non-nested clades, and so, individuals that co-cluster must belong to the same

transmission chain. Clustering algorithms therefore identify, first and foremost, distinct transmission chains, that are then termed "transmission clusters" if they satisfy certain criteria, usually a small enough genetic distance between their members and a minimum confidence level for their estimation. In other words, we can use standard phylogenetic clustering algorithms to find transmission chains: the only difference is in the requirements for the clades. In this paper, the terms *partitioning* and *clustering* are synonymous, and we use the term "transmission chain".

Confidence thresholds for transmission chain inference

Phylogenetic studies for the inference of transmission chains in HIV-1 epidemics have relied mostly on methods deemed *nonparametric*, as they tend to depend on a number of *ad hoc* rules applied a posteriori to phylogenetic estimates [14]. In particular, availability of software like MEGA and PAUP* [15, 16] has led to widespread adoption of maximum likelihood phylogenetic reconstruction, coupled with the bootstrap to evaluate confidence in the inferred clades. The scheme involves repeated resampling of site indices, and construction of simulated sequences based on the indices obtained. A phylogeny is then fitted to each simulated sample, and clades are listed. The proportion of times each clade appears in the obtained phylogenies is computed, which serves as the previously-mentioned measure of confidence. In that context, chain estimation depends on an arbitrary cutoff applied to bootstrap support estimates, usually between 70% and 95% [11, 17, 18].

Alternatively, software like BEAST and MrBayes [19, 20] have popularised Bayesian phylogenetic estimation. Both are based on versions of the Markov Chain Monte Carlo (MCMC) algorithm, that numerically approximate posterior distributions for a variety of evolutionary and phylogenetic parameters. They also provide posterior probability support for clades, a Bayesian alternative to bootstrap support. Most of the times, studies require posterior probability support of 1 to conclude in a clade forming a genuine transmission chain [21, 22].

A popular alternative to both bootstrap-based and Bayesian estimates of clade support is the approximate likelihood ratio test (aLRT) for branches [23], more specifically the aLRT-SH non-parametric branch support estimate. It is available in

PhyML [24] and IQtree [25]. It consists in a test statistic that indicates to what extent a given branch contributes to a gain in the phylogenetic likelihood, in comparison to the case where its length is reduced to zero, thus eliminating it altogether.

Distance requirements for transmission chain inference

In addition to clade confidence requirements, studies often impose a within-chain genetic distance requirement, usually between 0.01 nt/bp and 0.05 nt/bp [26]. Distance requirements may be applicable to mean [8], median [27], or maximum *patristic* distances [11], also called tree or *cophenetic* distances, that is, distances calculated by summing branch lengths along the shortest path between any two tips in the phylogeny. The *ClusterPicker* algorithm instead formulates that requirement in terms of maximum within-chain p-distances, e.g. the Hamming distance [26]. Both p-distances and patristic distances are measures of genetic distance, with the former being computed separately for each pair of sequences, and the latter being based on information obtained from the whole sample [22]. As noted previously, sets of sequences that meet both the confidence and distance requirements are usually termed "transmission clusters".

Summarising samples of trees

Both the bootstrap and the MCMC algorithms produce samples of trees that must be summarised before the application of confidence or genetic distance criteria. One strategy involves using the maximum likelihood (ML) or maximum posterior probability (MAP) tree, and applying criteria solely to the clades they contain. However, it is common for phylogenetic estimation procedure to produce several trees with likelihood or posterior probability very close to the maximum. The data may therefore support a wide variety of phylogenies, and this is not properly reflected by the ML or MAP trees. To address the issue, a majority-rule consensus tree can be constructed instead: in it, bifurcations support clades found in more than 50% of sampled trees; otherwise, multifurcations are used [28]. The majority-rule consensus tree can be shown to always exist, but it lacks branch lengths [22]. In other words, it provides only a nesting order for clades, which precludes the application of patristic

distance requirements.

Alternative partitioning approches

Cutoffs are however hard to justify rigorously [12] and so, methods grounded in more explicit definitions of chains have been published. For example, [29] proposed the so-called *Gap Procedure*, a fast pure distance-based approach that requires minimal tuning. Indeed, it involves a single tuning parameter, whose purpose is to eliminate the effect of outliers on estimation and whose value rarely need to be changed. In a similar vein, [30] formulated DM-PhyClus, a Bayesian algorithm that aims to limit reliance on hard thresholds and to offer a straightforward measure of uncertainty for estimates of chain membership. Other options are also available [31–33].

Computational challenges

The heavy computational burden of conventional phylogenetic inference is problematic in light of the fast increase in the size of sequence databases, and can therefore restrict its applicability [34]. Thankfully, software designed to handle larger datasets is now available. RAxML [35] and FastTree [36], for example, make use of heuristics in phylogenetic optimisation to improve scalability of the maximum likelihood phylogenetic methods. Partitioning of large datasets in a purely Bayesian paradigm is a computational challenge that has not yet been fully overcome, although vast progress has been made thanks in part to GPU computing [19, 20].

References

1. Dudas G, Rambaut A. Phylogenetic analysis of Guinea 2014 EBOV Ebolavirus outbreak. PLoS Currents. 2014;6.

2. Kenah E, Britton T, Halloran ME, Longini IM Jr. Molecular Infectious Disease Epidemiology: Survival Analysis and Algorithms Linking Phylogenies to Transmission Trees. PLOS Comput Biol. 2016;12(4):1–29. doi:10.1371/journal.pcbi.1004869.

3. Foley BT, Leitner TK, Apetrei C, Hahn B, Mizrachi I, Mullins J, et al. HIV Sequence Compendium 2015. Los Alamos National Lab (LANL), Los Alamos, NM (United States); 2015.

4. Huerta-Cepas J, Capella-Gutiérrez S, Pryszcz LP, Marcet-Houben M, Gabaldón T. PhylomeDB v4: zooming into the plurality of evolutionary histories of a genome. Nucleic Acids Res. 2014;42(Database issue):D897–D902. doi:10.1093/nar/gkt1177.

5. Goodreau SM, Carnegie NB, Vittinghoff E, Lama JR, Sanchez J, Grinsztejn B, et al. What drives the US and Peruvian HIV epidemics in men who have sex with men (MSM)? PLOS ONE. 2012;7(11):e50522.

6. Swiss HIV Cohort Study. Cohort profile: the Swiss HIV Cohort study. Int J Epidemiol. 2010;39(5):1179–1189. doi:10.1093/ije/dyp321.

7. Foley BT, Korber BTM, Leitner TK, Apetrei C, Hahn B, Mizrachi I, et al. HIV Sequence Compendium 2018. Los Alamos National Lab (LANL), Los Alamos, NM (United States); 2018.

8. Hué S, Clewley JP, Cane PA, Pillay D. HIV-1 pol gene variation is sufficient for reconstruction of transmissions in the era of antiretroviral therapy. AIDS. 2004;18(5):719–728.

9. Brenner BG, Wainberg MA. Future of Phylogeny in HIV Prevention. J Acquir Immune Defic Syndr. 2013;63 Suppl 2:S248–S254. doi:10.1097/QAI.0b013e3182986f96.

10. Brenner B, Wainberg MA, Roger M. Phylogenetic inferences on HIV-1 transmission: implications for the design of prevention and treatment interventions. AIDS. 2013;27(7):1045–1057. doi:10.1097/QAD.0b013e32835cffd9.

11. Brenner BG, Roger M, Routy JP, Moisi D, Ntemgwa M, Matte C, et al. High rates of forward transmission events after acute/early HIV-1 infection. J Infect Dis. 2007;195(7):951–959.

12. Chalmet K, Staelens D, Blot S, Dinakis S, Pelgrom J, Plum J, et al. Epidemiological study of phylogenetic transmission clusters in a local HIV-1

epidemic reveals distinct differences between subtype B and non-B infections. BMC Infect Dis. 2010;10:262. doi:10.1186/1471-2334-10-262.

13. Villandre L, Stephens DA, Labbe A, Günthard HF, Kouyos R, Stadler T, et al. Assessment of Overlap of Phylogenetic Transmission Clusters and Communities in Simple Sexual Contact Networks: Applications to HIV-1. PLoS One. 2016;11(2):e0148459.

14. Poon AF. Impacts and shortcomings of genetic clustering methods for infectious disease outbreaks. Virus Evol. 2016;2(2):vew031.

15. Tamura K, Stecher G, Peterson D, Filipski A, Kumar S. MEGA6: Molecular Evolutionary Genetics Analysis version 6.0. Mol Biol Evol. 2013;30(12):2725–2729. doi:10.1093/molbev/mst197.

16. Swofford DL. PAUP*: Phylogenetic analysis using parsimony (and other methods).; 2003. Available from: `http://paup.sc.fsu.edu/about.html`.

17. Hillis DM, Bull JJ. An Empirical Test of Bootstrapping as a Method for Assessing Confidence in Phylogenetic Analysis. Syst Biol. 1993;42(2):182–192. doi:10.1093/sysbio/42.2.182.

18. Holder M, Lewis PO. Phylogeny estimation: traditional and Bayesian approaches. Nat Rev Genet. 2003;4(4):275–284. doi:10.1038/nrg1044.

19. Drummond AJ, Suchard MA, Xie D, Rambaut A. Bayesian phylogenetics with BEAUti and the BEAST 1.7. Mol Biol Evol. 2012;29(8):1969–1973. doi:10.1093/molbev/mss075.

20. Ronquist F, Teslenko M, van der Mark P, Ayres DL, Darling A, Höhna S, et al. MrBayes 3.2: Efficient Bayesian Phylogenetic Inference and Model Choice Across a Large Model Space. Syst Biol. 2012;61(3):539–542. doi:10.1093/sysbio/sys029.

21. Erixon P, Svennblad B, Britton T, Oxelman B. Reliability of Bayesian Posterior Probabilities and Bootstrap Frequencies in Phylogenetics. Syst Biol. 2003;52(5):pp. 665–673.

22. Yang Z. Computational Molecular Evolution. Oxford Series in Ecology and Evolution. Oxford: Oxford University Press; 2006.

23. Anisimova M, Gascuel O. Approximate Likelihood-Ratio Test for Branches: A Fast, Accurate, and Powerful Alternative. Systematic Biology. 2006;55(4):539–552. doi:10.1080/10635150600755453.

24. Guindon S, Dufayard JF, Lefort V, Anisimova M, Hordijk W, Gascuel O. New algorithms and methods to estimate maximum-likelihood phylogenies: assessing the performance of PhyML 3.0. Systematic biology. 2010;59(3):307–321.

25. Nguyen LT, Schmidt HA, von Haeseler A, Minh BQ. IQ-TREE: a fast and effective stochastic algorithm for estimating maximum-likelihood phylogenies. Molecular biology and evolution. 2014;32(1):268–274.

26. Ragonnet-Cronin M, Hodcroft E, Hué S, Fearnhill E, Delpech V, Brown AJL, et al. Automated analysis of phylogenetic clusters. BMC Bioinformatics. 2013;14:317.

27. Prosperi MCF, Ciccozzi M, Fanti I, Saladini F, Pecorari M, Borghi V, et al. A novel methodology for large-scale phylogeny partition. Nat Commun. 2011;2:321.

28. Larget B, Simon DL. Markov chain Monte Carlo algorithms for the Bayesian analysis of phylogenetic trees. Mol Biol Evol. 1999;16:750–759.

29. Vrbik I, Stephens DA, Roger M, Brenner BG. The Gap Procedure: for the identification of phylogenetic clusters in HIV-1 sequence data. BMC Bioinformatics. 2015;16:355. doi:10.1186/s12859-015-0791-x.

30. Villandré L, Labbe A, Brenner B, Roger M, Stephens DA. DM-PhyClus: a Bayesian phylogenetic algorithm for infectious disease transmission cluster inference. BMC Bioinformatics. 2018;19(1):324.

31. Hassan AS, Pybus OG, Sanders EJ, Albert J, Esbjörnsson J. Defining HIV-1 transmission clusters based on sequence data. AIDS. 2017;31(9):1211.

32. Patiño-Galindo JÁ, Torres-Puente M, Bracho MA, Alastrué I, Juan A, Navarro D, et al. Identification of a large, fast-expanding HIV-1 subtype B transmission cluster among MSM in Valencia, Spain. PLOS ONE. 2017;12(2):e0171062.

33. Sallam M, Esbjörnsson J, Baldvinsdóttir G, Indridason H, Björnsdóttir TB, Widell A, et al. Molecular epidemiology of HIV-1 in Iceland: Early introductions, transmission dynamics and recent outbreaks among injection drug users. Infect Genet Evol. 2017;49:157–163.

34. Wang Y, Yang Z. Priors in Bayesian phylogenetics. Bayesian phylogenetics: methods, algorithms, and applications Chapman and Hall/CRC. 2014; p. 5–23.

35. Stamatakis A. RAxML Version 8: A tool for Phylogenetic Analysis and Post-Analysis of Large Phylogenies. Bioinformatics. 2014;doi:10.1093/bioinformatics/btu033.

36. Price MN, Dehal PS, Arkin AP. FastTree 2 – Approximately Maximum-Likelihood Trees for Large Alignments. PLOS One. 2010;5(3):1–10. doi:10.1371/journal.pone.0009490.
