# Supplementary material for: Assessing the role of transmission chains in the spread of HIV-1 among men who have sex with men in Quebec, Canada
Source: PLoS One. 2019 Mar 6;14(3):e0213366. doi: 10.1371/journal.pone.0213366 (PMC6402664; doi:10.1371/journal.pone.0213366)
Supplement: S2 Text — (PDF) [file pone.0213366.s002.pdf]

Supplementary Material S2: Cutpoint selection with a partial gold standard

The reference set includes only a small fraction of sequences in the dataset, and acts therefore as a *partial gold standard*. We select cutpoints for each method as to maximise overlap with that reference set. The lack of a reference solution for other sequences in the sample makes comparison with this standard non-straightforward. Let us assume we have a sample of size 10, and that sequences 1-3 and 4-6 form two confirmed chains, labelled 1 and 2, respectively. A representation for chain membership in the full gold standard would be [1, 1, 1, 2, 2, 2, Not 1 or 2, Not 1 or 2, Not 1 or 2, Not 1 or 2]. To best quantify overlap with the full gold standard, in all partitions we test, all sequences that do not co-cluster with any element in the reference set are given a membership index equal to (Number of chains found among sequences in the reference set + 1). The full gold standard is reformulated in such a way that all sequences outside the reference set are given index (Number of true chains in the reference set + 1). In the example, the gold standard would be reformulated [1, 1, 1, 2, 2, 2, 3, 3, 3, 3]. Let's say a clustering algorithm returns configuration [1, 1, 2, 3, 3, 3, 3, 4, 4, 5]. To obtain the correct Adjusted Rand Index, we would need to transform it into [1, 1, 2, 3, 3, 3, 3, 4, 4, 4].
