# Supplementary material for: Assessing the role of transmission chains in the spread of HIV-1 among men who have sex with men in Quebec, Canada
Source: PLoS One. 2019 Mar 6;14(3):e0213366. doi: 10.1371/journal.pone.0213366 (PMC6402664; doi:10.1371/journal.pone.0213366)
Supplement: S3 Text — (PDF) [file pone.0213366.s003.pdf]

Supplementary Material S3: MrBayes script

```
begin mrbayes;
set autoclose=yes nowarn=yes;
execute brennerCompleteData.nex;
lset nst=6 rates=invgamma;
outgroup AB254141;
set beaglescaling=dynamic beaglesse=yes;
mcmc nruns=2 nchains=4 ngen=3000000 samplefreq=500
diagnfreq=10000 printfreq=500 append=yes;
sump relburnin=yes burninfrac=0.25;
end;
```
