# Supplementary material for: Assessing the role of transmission chains in the spread of HIV-1 among men who have sex with men in Quebec, Canada
Source: PLoS One. 2019 Mar 6;14(3):e0213366. doi: 10.1371/journal.pone.0213366 (PMC6402664; doi:10.1371/journal.pone.0213366)
Supplement: S4 Text — (PDF) [file pone.0213366.s004.pdf]

Supplementary Material S4: Tuning parameters used in the DM-PhyClus and Gap Procedure analyses

DM-PhyClus

- Number of discrete states for the within-chain and between-chain transition probability matrices: 20,
- Number of samples used to obtain transition probability matrices: $100,000$,
- Radius around mean within-chain and between-chain branch length estimates: 25%,
- Discrete gamma distribution parameter: 1,
- Bootstrap and distance requirements for initial chain estimate: 90%, 0.045,
- Limiting probabilities: $(A = 0.38, T = 0.24, C = 0.16, G = 0.21)$,
- Rate matrix Q:

$$\begin{bmatrix} -0.8891 & 0.0659 & 0.1324 & 0.6908 \\ 0.1047 & -0.7205 & 0.5477 & 0.0681 \\ 0.3096 & 0.8069 & -1.1801 & 0.0636 \\ 1.2540 & 0.0779 & 0.0494 & -1.3812 \end{bmatrix}$$

- Shape parameter for concentration parameter prior: 500,
- Scale parameter for concentration parameter prior: 0.2,
- Poisson rate for weight applied to the chain membership vector prior: 2368,
- Number of iterations: $220,000$.

Gap Procedure

- Threshold for largest gap search: 90%.
