# Supplementary material for: Assessing the role of transmission chains in the spread of HIV-1 among men who have sex with men in Quebec, Canada
Source: PLoS One. 2019 Mar 6;14(3):e0213366. doi: 10.1371/journal.pone.0213366 (PMC6402664; doi:10.1371/journal.pone.0213366)
Supplement: S5 Text — (PDF) [file pone.0213366.s005.pdf]

Supplementary Material S5: The linkage estimate

We obtain the linkage estimate by first projecting each chain membership vector produced by DM-PhyClus as an unweighted undirected network graph, where each sequence is represented by a vertex, and an edge between any two vertex implying co-clustering between the corresponding sequences. For example, chain membership vector [1, 1, 1, 2, 2, 2] would translate as a graph with six vertices, split between two disjoint components, each of those being a *fully-connected* graph. In other words, all vertices within each component are inter-connected. We can express an unweighted undirected network graph with an *adjacency matrix*, a symmetric matrix with as many rows and columns as vertices, with a 1 (0) at position (i, j) indicating a connection (no connection) between vertices i and j. Elements on the diagonal are set to 0.

Once we have adjacency matrices for all chain membership states visited by the chain, we average all the matrices element-wise, resulting in an adjacency matrix for a *weighted* undirected network. Values in that matrix, all between 0 and 1, indicate the strength of the association between any two sequences. We then run the *walktrap algorithm* on the corresponding graph to identify *communities* [**?**]. Communities are sets of vertices that are a lot more interconnected than would be expected from chance alone. The walktrap algorithm works by performing a large number of short random walks on the graph. It starts at a random vertex, and jumps to neighbouring vertices a fixed number of times. It is based on the principle that a short random walk starting in a community is more likely to end up in the same community, because of the high degree of interconnectedness between its vertices. The algorithm then outputs an estimate of community structure in the form of a vector of arbitrary community labels, which corresponds to the desired linkage estimate.
