# Supplementary material for: Assessing the role of transmission chains in the spread of HIV-1 among men who have sex with men in Quebec, Canada
Source: PLoS One. 2019 Mar 6;14(3):e0213366. doi: 10.1371/journal.pone.0213366 (PMC6402664; doi:10.1371/journal.pone.0213366)
Supplement: S6 Text — (PDF) [file pone.0213366.s006.pdf]

Supplementary Material S6: Additional bar plots depicting chain growth between January 1st, 2012 and February 1st, 2016

The bar plots in this section can be read like Figures 3 and 4. The labels at the end of each bar indicate the sequence collection dates for the first and last “recent” PHIs in the chain, that is, recorded on or after July 1st, 2012. When there is only one such PHI, we display the corresponding collection date instead. We assume that all chronic cases recorded before July 1st, 2012 were infected prior to 2012. The dark red bar represents the “minimum chain size before 2012” because several chronic cases diagnosed after July 1st 2012 were probably infected prior to 2012. Also, it is likely that several PHIs sampled in the first half of 2012 match with transmission events that occurred late in 2011.

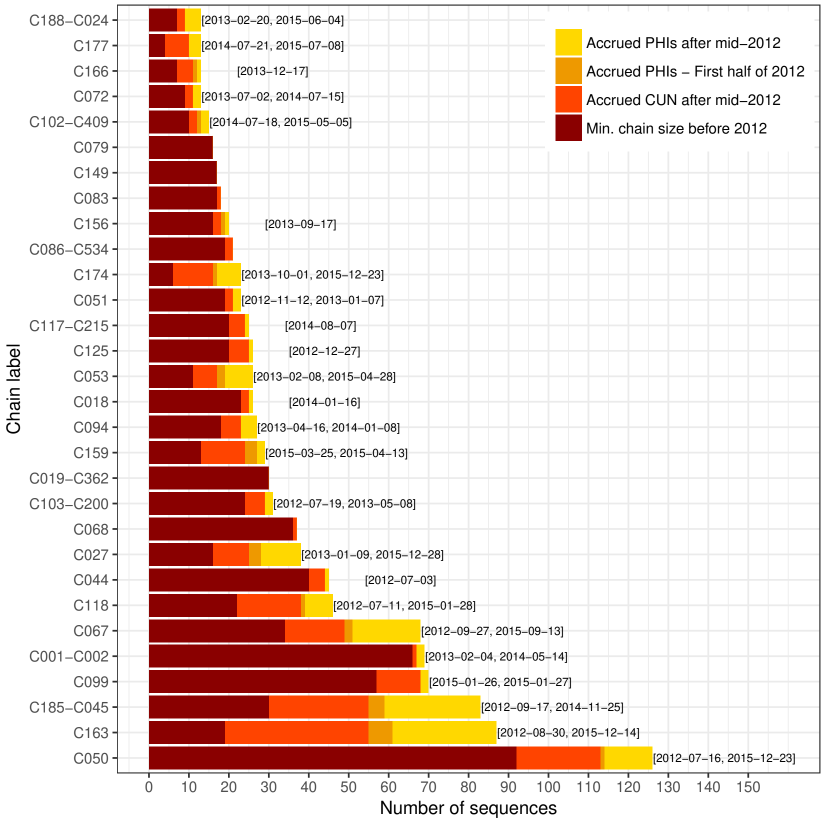

Fig 1. Bar plot showing the breakdown in membership for the 30 largest chains in the ML + ClusterPicker estimate.

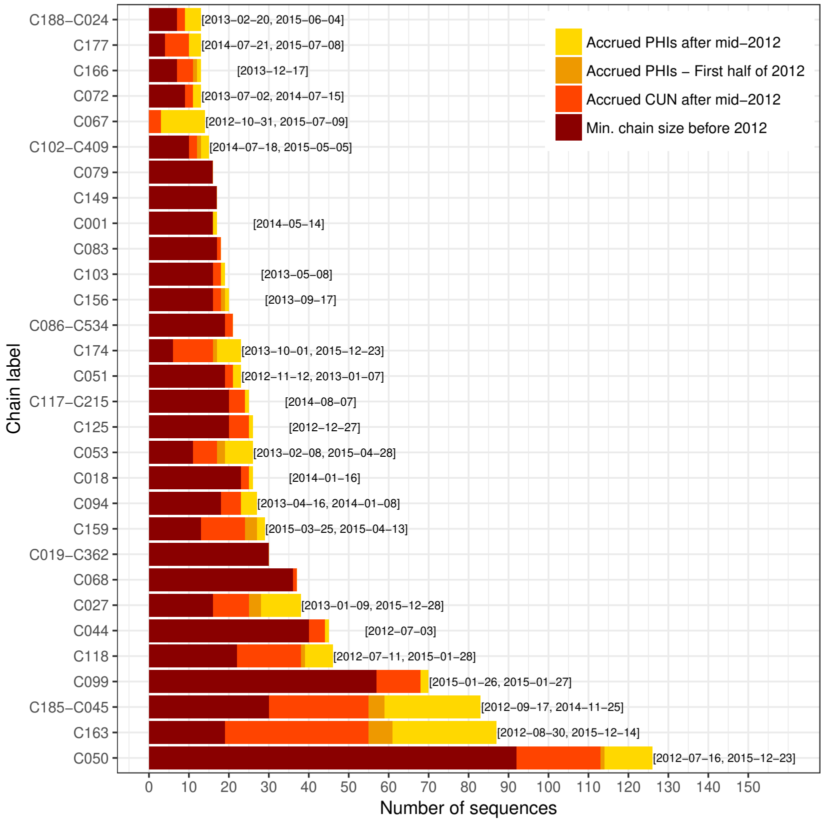

Fig 2. Bar plot showing the breakdown in membership for the 30 longest chains in the ML + maximum patristic distance estimate.

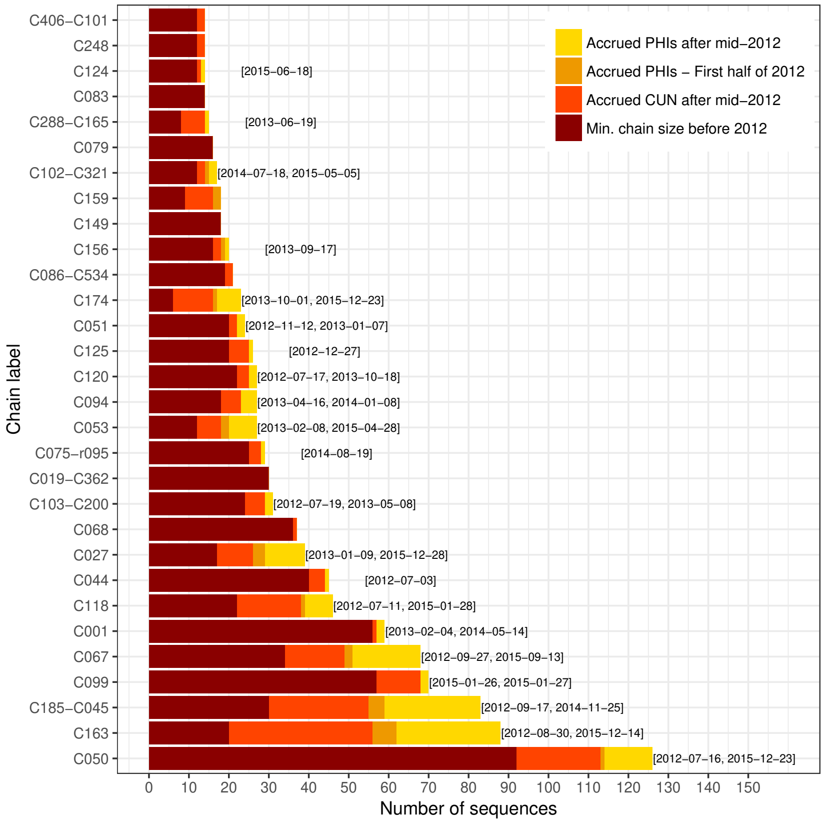

Fig 3. Bar plot showing the breakdown in membership for the 30 longest chains in the MrBayes+CP estimate.

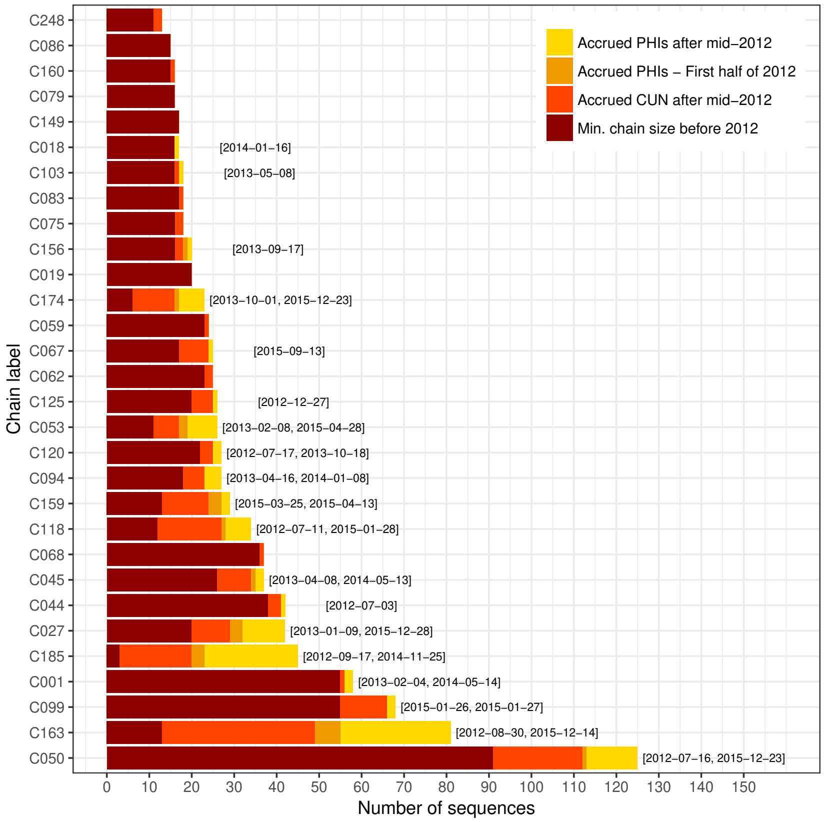

Fig 4. Bar plot showing the breakdown in membership for the 30 longest chains in the Gap Procedure estimate.

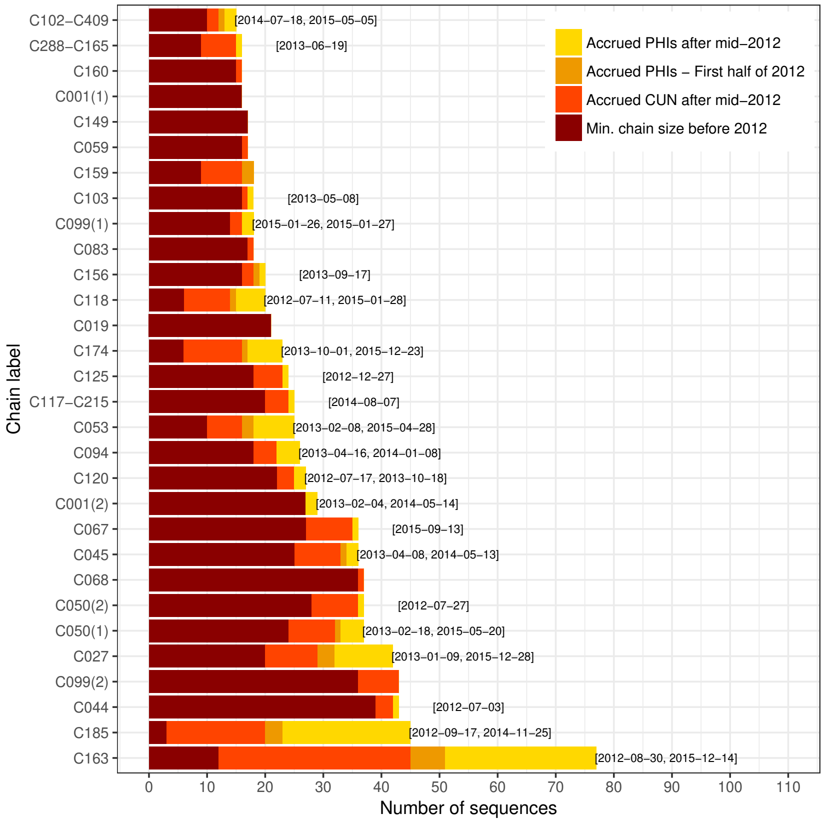

Fig 5. Bar plot showing the breakdown in membership for the 30 longest chains in the Gap Procedure estimate.
